# UFMylation promotes orthoflavivirus infectious particle production

**DOI:** 10.1128/jvi.00654-25

**Published:** 2025-06-03

**Authors:** Hannah M. Schmidt, Grace C. Sorensen, Matthew R. Lanahan, Katherine M. Bland, Caroline J. Aufgebauer, Moonhee Park, Jenna Grabowski, Stacy M. Horner

**Affiliations:** 1 Department of Molecular Genetics and Microbiology, Duke University School of Medicine 12277 , Durham, North Carolina, USA; 2 Department of Integrative Immunobiology, Duke University School of Medicine 12277 , Durham, North Carolina, USA; 3 Department of Medicine, Duke University School of Medicine 12277 , Durham, North Carolina, USA; Wake Forest University School of Medicine, Winston-Salem, North Carolina, USA

**Keywords:** orthoflavivirus, flavivirus, UFM1, UFMylation, ubiquitination, post-translational modification, PTM

## Abstract

Post-translational modifications play crucial roles in regulating viral infections, yet roles for many modifications remain unexplored in orthoflavivirus biology. Here, we demonstrate that the UFMylation system, a post-translational modification pathway that catalyzes the transfer of UFM1 onto proteins and promotes infection by multiple orthoflaviviruses, including dengue virus (DENV), Zika virus (ZIKV), West Nile virus, and yellow fever virus. We found that depletion of the UFMylation E3 ligase complex proteins UFL1 and UFBP1, as well as other UFMylation machinery components (UBA5, UFC1, and UFM1), significantly reduces orthoflavivirus infectious virion production. This regulation was specific to orthoflaviviruses as the hepacivirus and member of the broader *Flaviviridae* family, hepatitis C virus, was not regulated by UFL1. Mechanistically, UFMylation did not regulate viral RNA translation, RNA replication, or virion egress but instead affected the assembly of infectious virions. Furthermore, we identified novel interactions between UFL1 and several viral proteins involved in orthoflavivirus virion assembly, including NS2A, NS2B-NS3, and capsid. These findings establish UFMylation as a previously unrecognized post-translational modification pathway that promotes orthoflavivirus infection through modulation of viral assembly. This work expands our understanding of the post-translational modifications that control orthoflavivirus infection and identifies new potential therapeutic targets.

**IMPORTANCE:**

Orthoflaviviruses depend on host-mediated post-translational modifications to successfully complete their life cycle, yet many of these critical interactions remain undefined. Here, we describe a role for a post-translational modification pathway, UFMylation, in promoting infectious particle production of ZIKV and DENV. We show that UFMylation is dispensable for initial RNA translation and RNA replication but promotes the assembly of infectious virions. Additionally, we find that regulation of infection by UFMylation extends to other orthoflaviviruses, including West Nile virus and yellow fever virus, but not to the broader *Flaviviridae* family. Finally, we demonstrate that UFMylation machinery directly interacts with specific DENV and ZIKV proteins during infection. These studies reveal a previously unrecognized role for UFMylation in regulating orthoflavivirus infection.

## INTRODUCTION

Orthoflaviviruses are a genus of positive-sense RNA viruses (1) that represent a significant human health burden. These viruses, which include dengue virus (DENV), West Nile Virus (WNV), yellow fever virus (YFV), and Zika virus (ZIKV), are transmitted by arthropods in tropical regions, placing billions of people at risk of contracting an orthoflavivirus infection annually (2). There is currently a lack of therapies and broadly effective vaccines against these viruses, highlighting the need for a better understanding of the molecular processes occurring during viral infection. Orthoflaviviruses have a compact but efficient genome organization and life cycle that enable successful viral infection. Within infected cells, the ~11 kb positive-sense RNA genome is translated as a single polyprotein that is cleaved by viral and host proteases into 10 individual proteins (3). These viral proteins include three structural proteins (C, prM/M, and E) which form the virion and seven non-structural proteins (NS1, NS2A, NS2B, NS3, NS4A, NS4B, and NS5) that mediate viral replication and coordinate additional functions that contribute to infection, such as facilitating evasion of the innate immune system (4). Following translation, the viral proteins induce endoplasmic reticulum (ER) invaginations to compartmentalize viral RNA replication (3). Then, the viral genomic RNA is transported from the ER invaginations to associate with the viral capsid (C) protein, forming the nucleocapsid (5). The viral nucleocapsid buds through the ER to form an immature virion which undergoes additional maturation before being secreted from the cell (3). Due to their limited genome size, orthoflaviviruses rely on host factors to dynamically regulate these distinct viral life cycle stages (6–8). While many roles for host proteins in orthoflavivirus infection have been characterized, the full scope of host factors regulating orthoflavivirus infection is unknown, including roles for many enzymes catalyzing post-translational modifications.

Viral infection can be regulated by reversible post-translational modifications (9), which modify viral or host proteins to alter their stability, subcellular localization, and function. While roles for some post-translational modifications of orthoflaviviral proteins, such as acetylation, phosphorylation, ubiquitination, and glycosylation, have been described (10–13), there are many post-translational modifications, including novel ubiquitin-like modifications, that have not been fully explored during orthoflavivirus infection. One such ubiquitin-like modification is UFM1. UFM1 is an 85-amino acid ubiquitin-like peptide conjugated onto lysine residues through an enzymatic pathway involving the E1 activating enzyme, UBA5, the E2 conjugating enzyme, UFC1, and the E3 scaffold-like ligase complex, UFL1-UFBP1, while UFSP2 mediates its removal (14–19). The addition of UFM1 to proteins, which is referred to as UFMylation, can regulate protein function by altering protein-protein interactions (20–22). UFMylation regulates several host processes essential to viral infection and can modulate infection by a number of diverse viruses, including the γ-herpesvirus Epstein-Barr virus (EBV) (23) and the picornavirus hepatitis A virus (HAV) (24), ultimately limiting inflammation or promoting viral translation during infection, respectively. UFL1 has also been described to regulate pathways that could impact orthoflavivirus infection, such as promoting antiviral RIG-I signaling (25) and resolving ER stress responses (20, 26, 27), which are known to accumulate during orthoflavivirus replication (28). However, a specific function for UFMylation during orthoflavivirus infection has not been described.

Here, we demonstrate that UFL1, the UFMylation machinery, and the process of UFM1 conjugation promote DENV and ZIKV infectious virion production by regulating viral assembly. Additionally, we find that UFL1 promotes infection of several orthoflaviviruses, including DENV, ZIKV, WNV, and YFV, but it does not regulate infection by the hepacivirus, hepatitis C virus (HCV). Mechanistically, we find that UFL1 can interact with the viral capsid, NS2A, and NS2B/NS3 proteins during orthoflavivirus infection, suggesting that UFL1 interactions with these viral proteins may regulate their function through direct modification or altered protein-protein interactions. These findings establish UFM1 and the process of UFMylation as post-translational regulators of orthoflavivirus infection.

## RESULTS

### The UFMylation E3 ligase complex proteins promote orthoflavivirus infection

**Fig 1:**
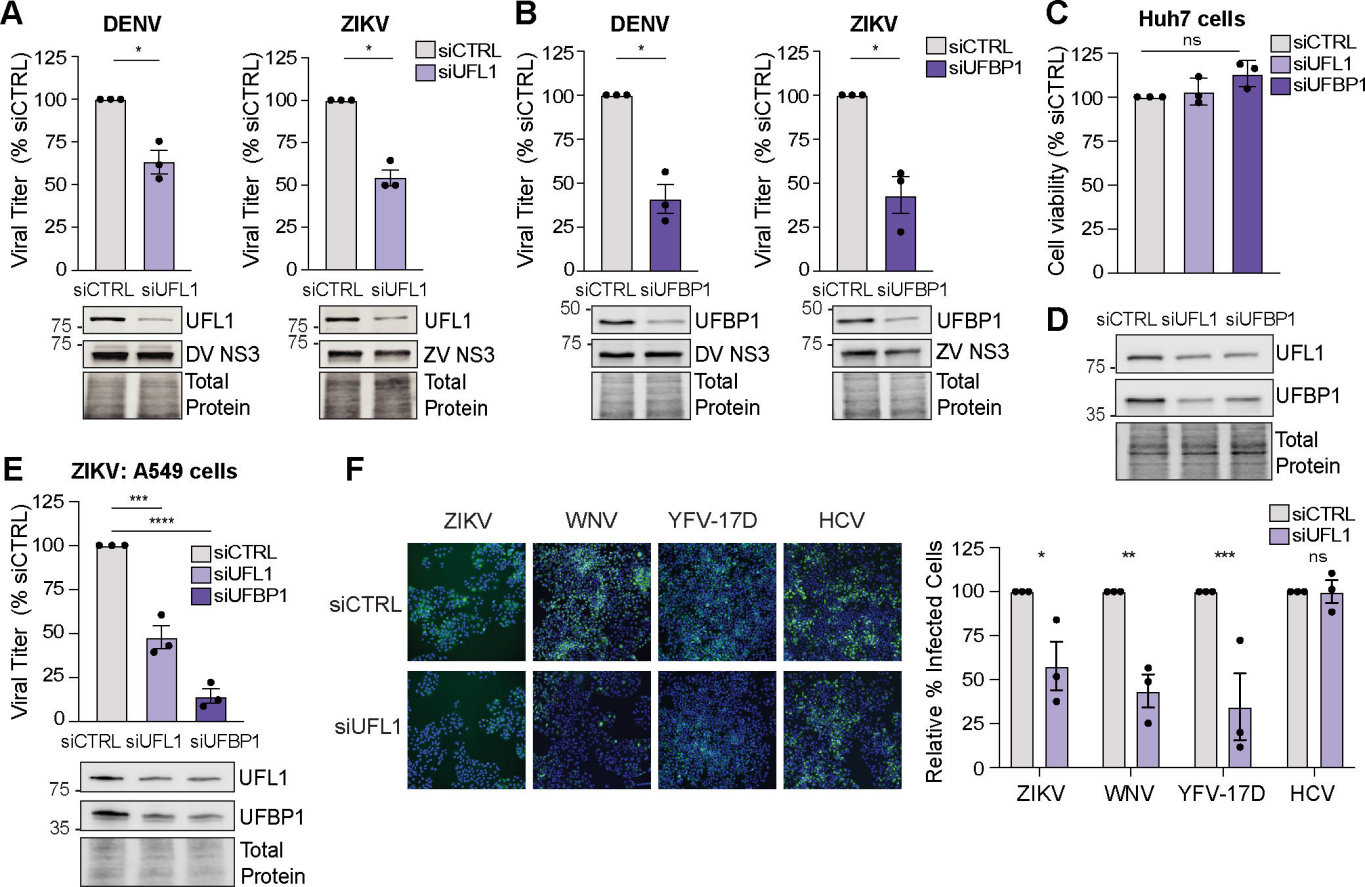
The UFMylation E3 ligase complex proteins promote mosquito-borne orthoflavivirus infection. (A and B) Focus-forming assay of supernatants from Huh7 cells infected with DENV^NGC^ or ZIKV^PRVABC59^ (48 h, MOI 0.1) after siRNA depletion of the indicated transcripts or non-targeting control (CTRL), shown as percentage of siCTRL. (**C**) Cell viability measured after siRNA depletion of the indicated transcripts at 72 h post-transfection, relative to that of siCTRL, as measured by Cell-Titer GLO assay. (**D**) Immunoblot analysis of protein expression from Huh7 cells treated with the indicated siRNAs for 72 h. (**E**) Focus-forming assay of supernatants harvested from A549 cells infected with ZIKV^PRVABC59^ (48 h, MOI 0.1) after siRNA depletion of the indicated transcripts. (**F**) Immunofluorescence micrographs of Huh7 cells treated with the indicated siRNA and then infected with the following viruses for 48 h: ZIKV^PRVABC59^, MOI 0.1; YFV^17D^, MOI 0.01; WNV^NY2000^, MOI 0.01, or HCV^JFH1^, MOI 1, as measured by immunostaining of viral antigens (E for ZIKV, YFV^17D^, and WNV, and NS5A for HCV; green). Nuclei were stained with Hoechst (blue). (Right) Quantification of the percentage of virus-infected Huh7 cells, shown relative to siCTRL with an average of 5,000 cells counted for each condition. For all panels, *n* = 3 biologically independent experiments, with bars indicating mean and error bars showing the standard error of the mean. *P < 0.05, **P < 0.01, ***P < 0.001, ****P < 0.0001, or not significant (ns) as determined by paired *t*-test (A and B), one-way analysis of variance (ANOVA) with Dunnett’s multiple comparison test (C and E), or two-way ANOVA followed by Šidák’s multiple comparison test (**F**).

To determine if the UFMylation E3 ligase complex protein UFL1 regulates orthoflavivirus infection, we examined the production of infectious virions during DENV or ZIKV infection following siRNA-mediated depletion of UFL1 in human hepatoma Huh7 cells. Huh7 cells are an appropriate model cell line for these viruses because they can support high levels of orthoflavivirus infection. In addition, orthoflavivirus infection induces disease pathologies associated with viral infection in the liver (29). We found that compared to cells treated with control non-targeting siRNA, depletion of UFL1 decreased the levels of infectious DENV in the supernatant at 48 h post-infection, as measured by focus-forming assay (FFA) (Fig. 1A, left). Similarly, depletion of UFL1 reduced the levels of infectious ZIKV (Fig. 1A, right). As recent studies have shown that the E3 ligase of UFMylation is a complex consisting of both UFL1 and UFBP1 (17, 30), we next examined the role of UFBP1 in DENV and ZIKV infection. We found that depletion of UFBP1 in Huh7 cells also resulted in decreased levels of infectious virions from both DENV and ZIKV infections, indicating that the UFMylation E3 ligase complex promotes orthoflavivirus infection (Fig. 1B). Importantly, depletion of UFL1 or UFBP1 did not affect the viability of Huh7 cells (Fig. 1C). However, the loss of expression of either UFL1 or UFBP1 in Huh7 cells did result in reduced expression of its cognate cofactor (Fig. 1D), as seen previously by others (17, 31). We observed similar results in A549 cells, a lung carcinoma line susceptible to orthoflavivirus infection, where depletion of either UFL1 or UFBP1 decreased the production of infectious ZIKV virions and resulted in reduced expression of both proteins (Fig. 1E). These results confirm that depletion of either UFL1 or UFBP1 results in loss of the overall UFMylation E3 ligase complex. In addition, these data reveal that depletion of the UFMylation E3 ligase complex results in reduced viral particle production during DENV and ZIKV infection. Moving forward, we solely targeted UFL1 expression to manipulate the expression of the UFMylation E3 ligase complex.

As the UFMylation E3 ligase complex was required to promote infection by both DENV and ZIKV, we next tested if it also regulated infection by other viruses in the *Flaviviridae* family. We depleted UFL1 by siRNA in Huh7 cells and measured the percentage of virus-infected cells during WNV, YFV^17D^, or HCV infection, using ZIKV as a control virus, as we have found it was regulated by UFL1. We found that UFL1 depletion resulted in a ~50% decrease in the percentage of cells infected by the orthoflaviruses ZIKV, WNV, and YFV-17D (Fig. 1F). However, UFL1 depletion had no effect on the percentage of cells infected by the hepacivirus HCV (Fig. 1F). Taken together, these data indicate that the UFMylation E3 ligase complex promotes infection of several orthoflaviviruses but not all viruses in the *Flaviviridae* family.

### The UFMylation E3 ligase complex does not regulate interferon induction during ZIKV infection

**Fig 2:**
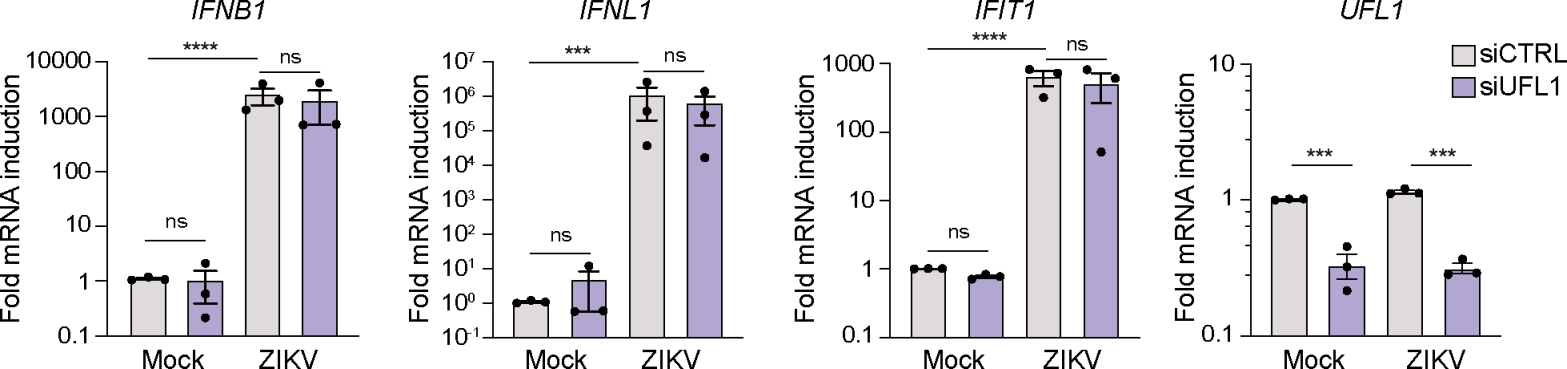
The UFMylation E3 ligase complex does not regulate interferon induction during ZIKV infection. RT-qPCR analysis (relative to *RPL30*) of RNA extracted from A549 cells transfected with either siCTRL or siUFL1 followed by mock or ZIKV^PRVABC59^ infection (24 h, MOI 0.1). *n* = 3 biologically independent experiments, with bars indicating the mean values and error bars showing the standard errors of the mean. ***P < 0.001, ****P < 0.0001, or not significant (ns), as determined by two-way ANOVA of log-transformed data followed by Šidák’s multiple comparisons test.

Since UFMylation can regulate interferon induction in response to RNA virus infection (25), we next wanted to determine if UFL1 regulated interferon induction during orthoflavivirus infection. To do this, we depleted UFL1 by siRNA in A549 cells, infected the cells with ZIKV, and then measured the induction of the *IFNB1*, *IFNL1*, and *IFIT1* transcripts by quantitative reverse transcription PCR (RT-qPCR). A549 cells are susceptible to orthoflavivirus infection and induce high levels of interferon in response to viral infections (32). Interestingly, while ZIKV induced *IFNB1*, *IFNL1*, and *IFIT1*, we found no difference in the induction of these transcripts upon depletion of UFL1 (Fig. 2). This suggests that UFL1 does not regulate orthoflavivirus infection by regulating interferon induction.

### The UFMylation E3 ligase complex does not regulate orthoflavivirus translation or RNA replication

**Fig 3:**
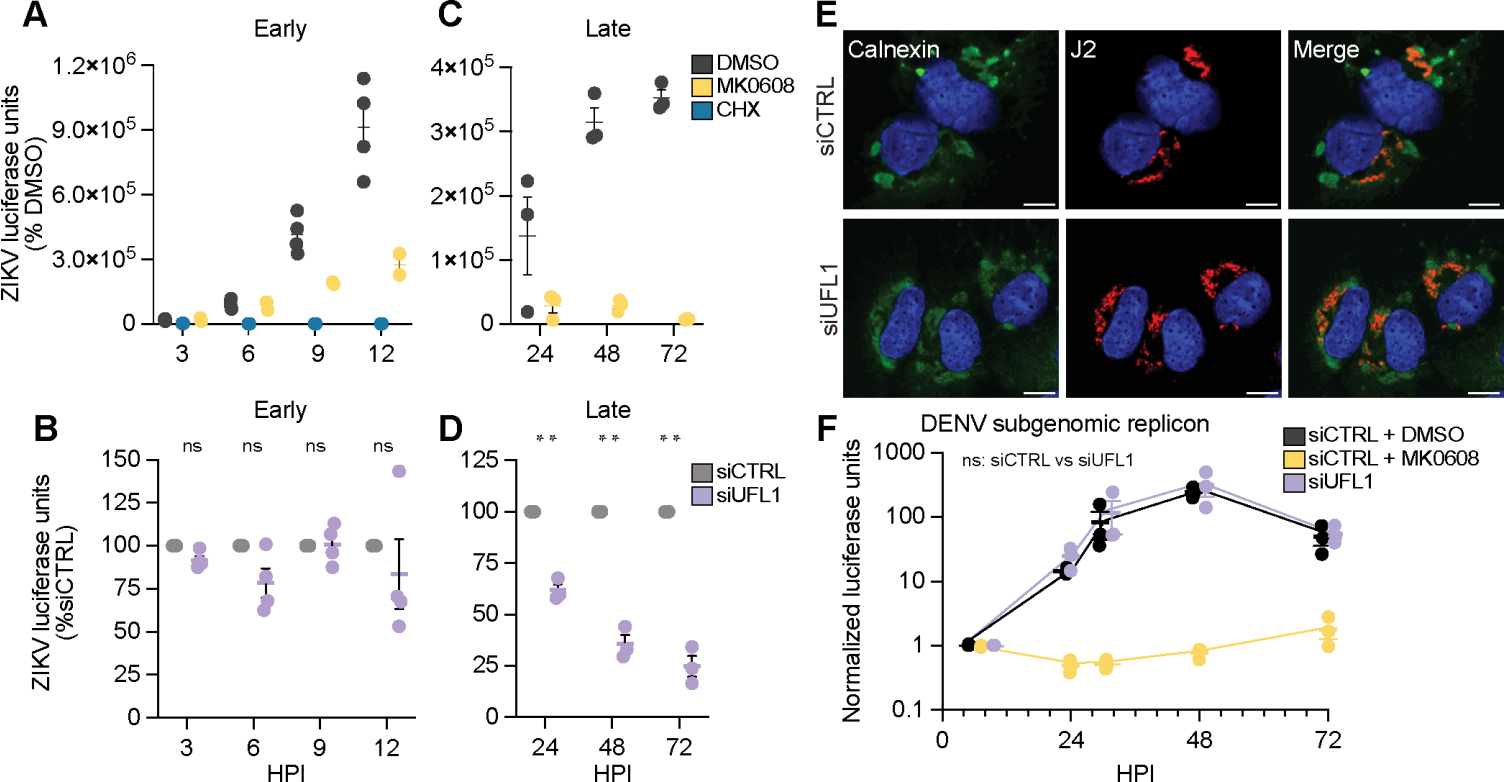
The UFMylation E3 ligase complex does not regulate orthoflavivirus translation or RNA replication. (**A**) Luciferase activity of *Gaussia* luciferase-encoding ZIKV^MR766^ (ZIKV-GLuc, MOI 0.1) from infected Huh7 cells treated with dimethyl sulfoxide (DMSO), MK0608, or cycloheximide during infection and harvested at the indicated time points. (**B**) Normalized expression of ZIKV-GLuc from infected Huh7 cells treated with non-targeting control (CTRL) or UFL1 siRNA harvested at the indicated time points. (**C**) Luciferase activity of *Gaussia* luciferase-encoding ZIKV^MR766^ (ZIKV-GLuc, MOI 0.1) from the supernatant of infected Huh7 cells treated with DMSO or MK0608 harvested at 24, 48, or 72 hpi. (**D**) Normalized luciferase activity of ZIKV-GLuc from the supernatant of infected Huh7 cells treated with CTRL or UFL1 siRNA and harvested at 24, 48, or 72 hpi. (**E**) Immunofluorescence micrographs of Huh7 cells treated with the indicated siRNA and then infected with ZIKV^PRVABC59^ (36 h, MOI 1) that were immunostained with anti-calnexin (green) and anti-J2 (red) for dsRNA, with the nuclei stained with Hoechst (blue). Scale bar, 10 µm. (**F**) Normalized luciferase expression of lysates from the expression of Huh7 cells transfected with the indicated siRNA and electroporated with a DENV^16681^ subgenomic RNA replicon expressing *Renilla* luciferase harvested at the indicated time points. Treatment with MK0608 was as in panel **A**. For all panels, *n* = 3 biologically independent experiments, with bars indicating mean and error bars showing standard error of the mean. **P < 0.01, or not significant (ns), determined by two-way ANOVA with Dunnett’s multiple comparison test (B, D, and F).

Having found that UFL1 and UFBP1 regulate orthoflavivirus infection, we next wanted to map the stage of the orthoflavivirus life cycle regulated by the UFMylation E3 ligase complex, using ZIKV and DENV as representative orthoflaviviruses. As others have shown that UFL1 and UFBP1 promote translation of the genome of the positive-strand RNA virus hepatitis A virus (24), we tested if UFL1 is required for ZIKV RNA translation. To do this, ecwe eff established time points corresponding to initial RNA tr tanslation and replication during DENV replicon with an infectious ZIKV reporter virus that encodes *Gaussia* luciferase (ZIKV-GLuc) (33), measuring *Gaussia* luciferase activity over a time course from 3 to 12 h post-infection. We detected *Gaussia* luciferase activity as early as 3 h post-infection, and this activity increased over the time course from 3 to 12 h, indicative of increased *Gaussia* luciferase expression (Fig. 3A). Importantly, cycloheximide treatment, which inhibits translation, resulted in decreased ZIKV-GLuc levels as early as 3 h post-treatment (Fig. 3A), while MK0608 treatment, which inhibits the viral RNA-dependent RNA polymerase (RdRp) (34), resulted in decreased ZIKV-GLuc levels at 9 h post-treatment (Fig. 3A). These results demonstrate that the signal observed from ZIKV-GLuc at 3 and 6 h is the product of viral translation alone and that following 9 h, viral RNA replication also contributes to the increasing levels of ZIKV-GLuc. Having established this system to measure the translation and replication of ZIKV, we next determined if UFL1 depletion alters these viral life cycle steps. To do this, we depleted UFL1 by siRNA in Huh7 cells, infected the cells with ZIKV-GLuc, and measured *Gaussia* luciferase expression over time. During 3 to 12 h post-infection, time points which (as we established) measure RNA translation and replication of ZIKV, depletion of UFL1 had no effect on the relative ZIKV-GLuc levels (Fig. 3B). However, depletion of UFL1 did reduce the levels of ZIKV-GLuc in the 24–72 h after infection (Fig. 3C and D). This suggests that UFL1 does not regulate viral RNA translation or RNA replication and instead acts on a later viral life cycle stage.

After the initial rounds of orthoflavivirus RNA translation, the viral proteins induce ER invaginations that compartmentalize viral RNA replication (35). As the UFMylation E3 ligase complex can regulate ER morphology (31), we next tested if UFL1 regulates the general morphology of the ER in ZIKV-infected Huh7 cells by examining the gross morphology of the characteristic viral dsRNA-containing ER membranes that accumulate in the perinuclear region, as seen by others (36, 37). Using immunofluorescence morphology with staining for the ER (calnexin) and dsRNA (J2), we found that depletion of UFL1 did not appear to broadly alter this morphology (Fig. 3E). To directly test if UFL1 regulates orthoflavivirus replication, we measured the replication of a subgenomic RNA replicon of DENV encoding a *Renilla* luciferase gene (DENV-RLuc-SGR) (8). This subgenomic RNA replicon lacks the viral structural genes but contains the non-structural genes sufficient for RNA replication, such that when *in vitro* transcribed RNA is transfected into cells, the viral RNA can replicate but cannot produce infectious virions. Following transfection of *in vitro* transcribed DENV-RLuc-SGR RNA into Huh7 cells, we found that UFL1 depletion did not alter *Renilla* luciferase activity levels over a time course, while the RdRp inhibitor MK0608, which inhibits RNA replication, did prevent RLuc expression, as expected (Fig. 3F). In summary, these data reveal that UFL1, and thus the UFMylation E3 ligase complex, promotes orthoflavivirus infection at a viral life cycle stage following RNA replication.

### The UFMylation E3 ligase regulates orthoflavivirus assembly

**Fig 4:**
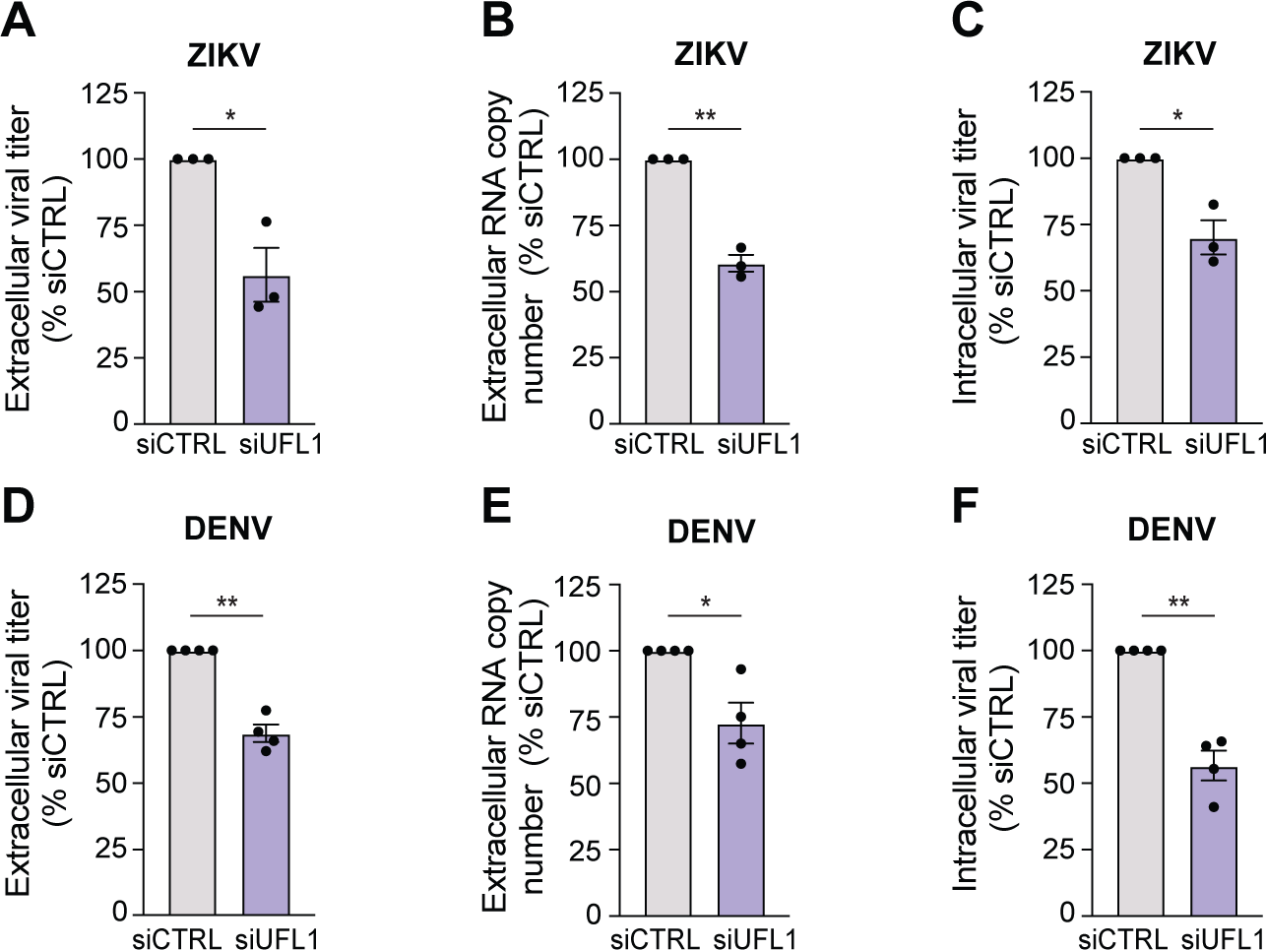
The UFMylation E3 ligase complex regulates assembly of intracellular infectious virions. (A–C) Huh7 cells were treated with the indicated siRNA and infected with ZIKV^PRVABC59^ for 48 h (MOI 0.1), and the following assays were performed: (**A**) focus-forming assay from supernatants to measure extracellular titer, (**B**) RNA copy number from supernatants, and (**C**) focus-forming assay from cellular lysates for intracellular titer. (D–F) Huh7 cells were treated with the indicated siRNA and infected with DENV^NGC^ for 48 h (MOI 0.1), and the following assays were performed: (**D**) focus-forming assay from supernatants for extracellular titer, (**E**) RNA copy number from supernatants, and (**F**) focus-forming assay from cellular lysates for intracellular titer. *n* = 3 (A–C) or *n* = 4 (D–F) biologically independent experiments, with bars indicating mean values and error bars showing standard errors of the mean. *P < 0.05, **P < 0.01, or not significant (ns), determined by paired *t*-test.

Having found that UFL1 depletion did not alter orthoflavivirus translation or RNA replication but did reduce infectious particle production, we next tested if UFL1 depletion affected viral assembly, viral release, or viral infectivity. To determine if viral infectivity is regulated by UFL1, we measured the extracellular titer and extracellular RNA of both ZIKV and DENV. Importantly, we validated our earlier results that UFL1 depletion results in decreased infectious virions secreted from the cell (extracellular titer) (Fig. 4A and D). In addition, we found that UFL1 depletion results in decreased viral RNA copy number in the supernatant (Fig. 4B and E), indicating that UFL1 depletion likely does not regulate the infectivity of extracellular virus particles. To determine if viral egress is regulated by UFL1, we measured the intracellular titer of both ZIKV and DENV. We found that UFL1 depletion results in decreased infectious virions produced within the cell for both ZIKV and DENV (Fig. 4C and F), indicating that UFL1 does not regulate viral egress. In summary, these data reveal that UFL1, and thus the UFMylation E3 ligase complex, does not promote viral egress but instead promotes the assembly of ZIKV and DENV virions.

### The UFMylation machinery promotes orthoflavivirus infection

**Fig 5:**
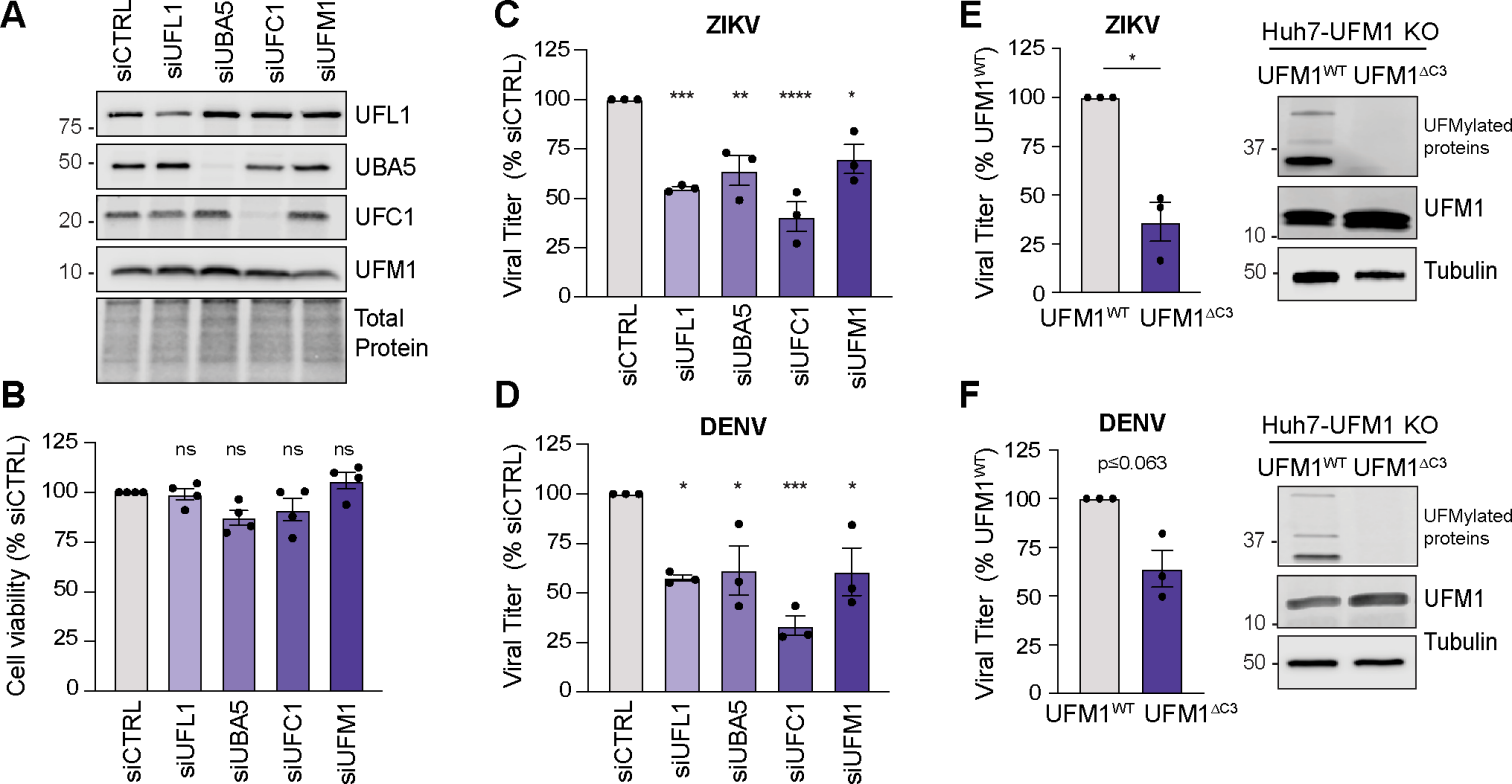
The UFMylation machinery promotes orthoflavivirus infection. (**A**) Immunoblot analysis of Huh7 cells after siRNA depletion of the indicated transcripts or non-targeting control (CTRL). (**B**) Cell viability measured after siRNA depletion of the indicated transcripts at 72 h post-transfection, as measured by Cell-Titer GLO assay, relative to the viability of siCTRL. (C and D) Focus-forming assay of supernatants harvested from Huh7 cells infected with DENV^NGC^ or ZIKV^PRVABC59^ (48 h, MOI 0.1) after siRNA depletion of the indicated transcripts, shown as percentage of siCTRL. (E and F) Focus-forming assay of supernatants harvested from Huh7-UFM1 KO cells transduced with Flag-UFM1^WT^ or Flag-UFM1^ΔC3^ and infected with either DENV^NGC^ (72 h, MOI 0.1) or ZIKV ^PRVABC59^ (48 h, MOI 0.1), shown as percentage of Flag-UFM1^WT^. Immunoblots indicate UFM1-conjugated proteins as those that are of higher molecular weight from unconjugated UFM1 but are detected with the anti-UFM1 antibody. For all panels, *n* = 3 biologically independent experiments, with bars indicating mean values and error bars showing standard errors of the mean. *P < 0.05, **P < 0.001, ***P < 0.001, ****P < 0.0001, or not significant (ns), determined by one-way ANOVA with Dunnett’s multiple comparison test (B, C, and D) or paired *t*-test (E and F).

Having found that the UFMylation E3 ligase complex promotes orthoflavivirus infection, we next wanted to determine if the other proteins that regulate UFMylation beyond the E3 ligase complex promote infection by DENV and ZIKV. To test this, we depleted the E1 activase UBA5, the E2 conjugase UFC1, or the ubiquitin-like modifier UFM1 in Huh7 cells using siRNA and then infected these cells with ZIKV and DENV. Importantly, we validated knockdown of the proteins by immunoblotting and confirmed that transient depletion of the UFMylation machinery did not affect cell viability of Huh7 cells compared to siCTRL as measured by Cell-Titer GLO assay (Fig. 5A and B). Depletion of each of the UFMylation machinery proteins reduced infectious virion production of ZIKV between ~35% and 65% compared to a non-targeting control (Fig. 5C). Similarly, depletion of the UFMylation machinery proteins reduced the infectious virion production of DENV (Fig. 5D). Importantly, we also validated our earlier results showing that depletion of UFL1 by siRNA resulted in reduced infectious virion production in either ZIKV or DENV (Fig. 5C and D). Since the complement of proteins involved in the process of UFM1 conjugation all positively regulate ZIKV and DENV infectious virion production, we next wanted to test if UFM1 conjugation itself is required to promote ZIKV and DENV infection. To do this, we transduced Huh7-UFM1 knockout (KO) cells that we generated by CRISPR/Cas9 with lentiviruses expressing Flag-tagged UFM1^WT^ or UFM1^∆C3^, in which the deletion of the last three residues of UFM1 prevented its conjugation to the lysine residues of target proteins (15). Importantly, we confirmed that UFM1^∆C3^ limits UFM1 conjugation, while UFM1^WT^ maintains UFM1 conjugation by measuring the formation of UFM1-conjugates by immunoblotting in Huh7-UFM1 KO cells complemented with Flag-UFM1^∆C3^ (Fig. 5E and F). When we infected the cells with ZIKV, we found that Huh7-UFM1 KO cells complemented with FLAG-UFM1^∆C3^ produced roughly half as many infectious virions as those cells complemented with FLAG-UFM1^WT^ (Fig. 5E). While the mean production of infectious DENV virions was lower in the Flag-UFM1^∆C3^ cells, this decrease was not statistically significant (Fig. 5F). Taken together, these data indicate that the UFMylation machinery proteins and UFM1 conjugation itself promote orthoflavivirus infection.

### UFL1 interacts with several DENV and ZIKV proteins

**Fig 6:**
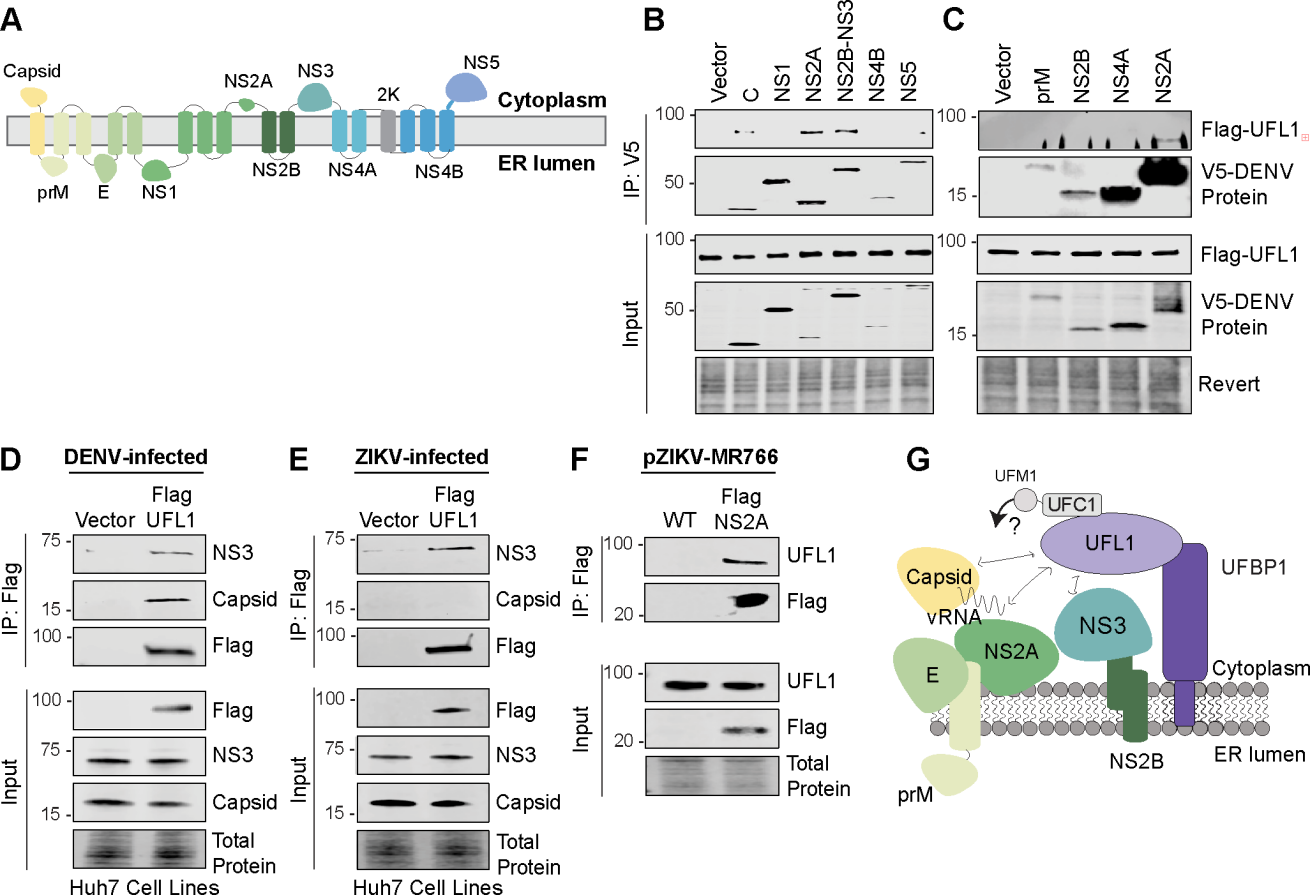
UFL1 interacts with several DENV and ZIKV proteins. (**A**) Schematic of DENV polyprotein, showing membrane topology of viral proteins. (B and C) Immunoblot analysis of anti-V5 immunoprecipitated extracts and inputs, lysed in NP40 buffer (**B**) or TX-100-RIPA buffer (**C**), from Huh7 cells stably expressing Flag-UFL1 transfected with plasmids expressing V5-tagged DENV^16681^ proteins. (D and E) Immunoblot analysis of anti-Flag immunoprecipitated extracts and inputs from DENV^NGC^-infected or ZIKV^PRVABC59^-infected (48 h, MOI 1) Huh7 cells stably expressing Flag-UFL1 or vector. (**F**) Immunoblot analysis of anti-Flag immunoprecipitated extracts and inputs from Huh7 cells transfected with DNA plasmids encoding the plasmid-launched ZIKV^MR766-WT^ or pZIKV ^MR766-Flag-NS2A^ and harvested at 72 hpi. Representative immunoblots from *n* = 3 biologically independent experiments are shown. (G) Model depicting UFL1 interactions wtih viral proteins.

Our results so far have revealed that UFL1 promotes DENV and ZIKV infectious particle production by regulating the assembly of infectious virions. Several host factors are known to promote orthoflavivirus assembly by interacting with viral proteins (5, 38–41). To uncover the mechanisms of how the UFMylation E3 complex regulates orthoflaviviral infectious virion production, we tested if UFL1 interacts with any of the orthoflaviviral proteins (Fig. 6A). We focused on UFL1 because, unlike UFBP1, it is not anchored to ER membranes (31), allowing us to more easily define protein-protein interactions between viral proteins and the UFMylation E3 complex in co-immunoprecipitation-based experiments. In an initial screen, we measured the interaction of UFL1 with a V5-tagged set of DENV proteins (42) by co-immunoprecipitation in Huh7 cells. For the experiments, we utilized different lysis conditions, depending on the viral protein. We found that DENV capsid, NS2A, and the NS2B-NS3 complex can interact with UFL1 in an overexpression setting (Fig. 6B). However, we did not detect interaction between UFL1 and prM, NS1, NS2B, NS4A, NS4B, or NS5 (Fig. 6B and C). Of note, the interaction of NS2A with UFL1 was detected regardless of the lysis buffer. We did not screen for interaction with viral E protein as we found that this construct did not express and is unlikely to interact with the cytosolic UFL1, as it is localized to the lumen of the ER (3). Thus, overexpression-based co-immunoprecipitation assays suggest interaction between UFL1 and three DENV proteins: Capsid, NS2A, and NS2B-NS3.

We next measured these protein-protein interactions in the context of viral infection. We infected Huh7 cells stably expressing Flag-UFL1 or Flag-tag alone with DENV or ZIKV and immunoprecipitated Flag-UFL1. During DENV infection, we found that both NS3 and capsid interacted with UFL1 (Fig. 6D). However, during ZIKV infection, we found that NS3, but not capsid, interacted with UFL1 (Fig. 6E). As no commercial antibodies are available for the orthoflaviviral NS2A protein, we sought to validate the interaction of UFL1 and NS2A during ZIKV infection using a ZIKV^Flag-NS2A^-expressing virus. This virus is similar to those generated by others, where a protein tag is cloned into the junction between NS1 and NS2A (43). In this case, a 3XFlag tag was inserted into the backbone of the plasmid-based rescue system for ZIKV MR766 (44). At 72 h post-transfection of pZIKV^Flag-NS2A^ or pZIKV^WT^, which does not contain any epitope tag on NS2A and served as our negative control, we immunoprecipitated Flag-NS2A using an anti-Flag antibody and found that it co-immunoprecipitated with endogenous UFL1 (Fig. 6F). Together, these data show that UFL1 interacts with specific DENV and ZIKV proteins during infection.

## DISCUSSION

Orthoflavivirus infection is a tightly coordinated process regulated by multiple mechanisms, including the localization of viral proteins to replication complexes, ER membrane rearrangements, and post-translational modifications on both viral and host proteins (3, 6, 10). While post-translational modifications such as acetylation, phosphorylation, ubiquitination, and glycosylation have all been shown to regulate various aspects of the orthoflavivirus life cycle by acting either on viral or host proteins (10–12, 45–47), our understanding of the full complement of regulatory modifications during orthoflavivirus infection remains incomplete. Here, we identify UFMylation as a novel post-translational modification system that positively regulates orthoflavivirus infection. We demonstrate that the UFMylation E3 ligase complex proteins, UFL1 and UFBP1, along with other components of the UFMylation machinery, promote infectious particle production for both DENV and ZIKV. Furthermore, we show that the UFMylation E3 ligase complex promotes infection by other orthoflaviviruses, including WNV and YFV, but does not regulate infection by the hepacivirus HCV. Mechanistically, our results reveal that UFMylation does not affect viral RNA translation, genomic RNA replication, or virion egress but instead regulates the assembly of infectious virions. Supporting this conclusion, we identify protein-protein interactions between UFL1 and orthoflaviviral proteins involved in virion assembly, specifically NS2A and NS2B-NS3 for DENV and ZIKV, and capsid for DENV. Taken together, our results highlight a novel role for UFMylation in promoting infectious virion production during orthoflavivirus infection.

The production of infectious orthoflavivirus particles relies on a precise sequence of viral and cellular protein interactions that direct distinct stages of the assembly process. During orthoflavivirus assembly, NS2A plays a key role in bringing the viral RNA genome from the replication complex to the virion assembly site at the ER membrane (48–50). At these sites, NS2A interacts with NS2B-NS3 to facilitate cleavage of capsid from the capsid-prM-E polyprotein (5, 51). This enables capsid dimerization and NS2A-mediated transfer of the genomic viral RNA to capsid, forming the viral nucleocapsid (5, 52, 53). The viral nucleocapsid then buds through the ER to form an immature virion coated with prM and E (5). While viral proteins mediate these steps, host proteins can regulate interactions important for virion assembly. One example of a host protein that regulates DENV assembly is YBX1, which promotes incorporation of the nucleocapsid into the virion (54). Another example is nucleolin, which interacts with capsid to promote virion assembly (38). Of the limited set of host proteins with roles in orthoflavivirus particle production, many function through interactions with viral proteins (38, 54, 55). This parallels our findings with UFL1, which interacts with viral assembly proteins and promotes assembly. We hypothesize that the UFMylation machinery coordinates specific assembly steps such as (i) NS2A-mediated RNA transfer from the replication complex to assembly sites, (ii) NS2B-NS3 processing of capsid, or (iii) capsid dimerization and RNA interaction. The absence of UFL1-capsid interaction in ZIKV (Fig. 6D) suggests virus-specific adaptations, possibly reflecting slight differences in capsid processing kinetics or assembly mechanisms between DENV and ZIKV (56). Future studies will be aimed at determining whether UFM1 directly modifies viral proteins or if it modifies host factors to regulate their roles in virion assembly.

The role of UFMylation in orthoflavivirus assembly contrasts with its regulatory functions in other viral systems, where it has been shown to regulate interferon induction, translation, and the NLRP3 inflammasome. For example, we have shown that UFMylation regulates RIG-I-mediated interferon induction upon RNA virus infection (25, 57). However, here we found that depletion of UFL1 did not significantly alter ZIKV-induced antiviral innate immune activation, suggesting that the proviral role of UFL1 in orthoflavivirus infection is independent of interferon signaling. This likely stems from orthoflaviviruses blocking the stage of RIG-I activation regulated by UFL1 (25, 58, 59). Beyond its role in RIG-I activation, UFMylation has been shown to regulate other host processes relevant to viral infection. During HAV infection, UFMylation of the ribosomal protein RPL26, located near the ribosome exit tunnel, is required for optimal viral translation (24). In contrast, during EBV infection, the viral protein BILF1 promotes the UFMylation of MAVS to facilitate its degradation via the lysosomal pathway to dampen the NLRP3 inflammasome (23). Our work reveals a distinct mechanism in which UFL1 regulates viral assembly and interacts with viral assembly proteins. This distinct finding underscores the versatility of UFMylation as a host pathway co-opted by viruses. For orthoflaviviruses, UFMylation may regulate protein-RNA interactions (e.g., capsid- or NS2A-RNA genome binding), specific viral protein-protein interactions that coordinate assembly (e.g., NS2B-NS3 with NS2A or capsid), or host factors that have roles in virion assembly, though the precise mechanisms remain to be elucidated.

Post-translational modifications have emerged as direct regulators of both viral and host proteins, regulating multiple aspects of the orthoflaviviral life cycle. K63-linked ubiquitination enhances ZIKV entry (12); SUMOylation stabilizes DENV NS5 (60); and now, we have shown here that UFMylation is an assembly-specific regulator of ZIKV and DENV. While UFMylation represents a promising avenue to explore for host-directed antiviral therapies, much remains to be learned about how the UFMylation machinery selects its targets during infection to minimize effects on host cell pathways that could lead to toxicity. Altogether, our study reveals a novel role for the process of UFMylation as a regulator of orthoflavivirus infection through modulation of virion assembly and broadens our understanding of the host factors required for successful viral replication.

## MATERIALS AND METHODS

### Cell culture

Huh7 cells, Huh7.5 cells, A549 cells, Vero cells, and 293T cells were grown in Dulbecco’s modification of Eagle’s medium (DMEM, Mediatech) supplemented with 10% fetal bovine serum (HyClone), 1× minimum essential medium non-essential amino acids (Thermo Fisher), and 25 mM HEPES (Thermo Fisher), referred to as complete DMEM (cDMEM). The identity of the Huh7 and Huh7.5 cells was verified by using the GenePrint STR kit (Duke DNA Analysis Facility). C6/36 cells were grown in Eagle’s minimum essential media (American Type Culture Collection [ATCC]) supplemented with 10% fetal bovine serum (HyClone), 25  mM N-2-hydroxyethylpiperazine-N′-2-ethanesulfonic acid (Thermo Fisher), and 1× non-essential amino acids (Thermo Fisher). Cells were obtained from the following sources: A549 cells, 293T, Vero cells, and C6/36 cells (CCL185, CRL-3216, CCL-81, and CCL-1660, respectively) from ATCC; Huh7 and Huh7.5 cells were from Dr. Michael Gale Jr. (61). All cell lines were verified as mycoplasma free by the MycoStrip Mycoplasma Detection Kit (InvivoGen).

### Plasmids

The following plasmids were generated by insertion of PCR-amplified fragments from DENV Open Reading Frames (gift of Dr. Priya Shah) (42) into the KpnI-to-BstBI digested pEF-TAK-V5 using InFusion (Clontech): pEF-TAK-DENV-C-V5, pEF-TAK-DENV-pRM-V5, pEF-TAK-DENV-pRM-E-V5, pEF-TAK-DENV-NS1-V5, pEF-TAK-DENV-NS2A-V5, pEF-TAK-DENV-NS2B-V5, pEF-TAK-DENV-NS3-V5, pEF-TAK-DENV-NS4A-V5, pEF-TAK-DENV-NS4B-V5, and pEF-TAK-DENV-NS5-V5. The following plasmids were generated by insertion of PCR-amplified fragments into the XbaI-to-BamHI digested pLVX vector (Clontech): pLVX-Flag and pLVX-Flag-UFL1. The following plasmids were generated by insertion of PCR-amplified fragments into the EcoRI-to-BamHI digested pLVX vector: pLVX-Flag-UFM1 and pLVX-Flag-UFM1ΔC3.

### Antibodies

For immunoblotting, the following primary antibodies were used: R-anti-UFL1 (Novus Biologicals; NBP1-79039, 1:1,000), R-anti-UFBP1 (DDRGK1, Proteintech; 21445-1-AP, 1:1,000), R-anti-UBA5 (Abcam; ab177478, 1:1,000), R-anti-UFC1 (Abcam; ab189252, 1:1,000), R-anti-UFM1 (Abcam; ab109305, 1:1,000), M-anti-DENV NS3 (GeneTex; GT2811, 1:1,000), R-anti-ZIKV NS3 (GeneTex; GTX133320, 1:1,000), R-anti-DENV capsid (GeneTex; GTX103343, 1:1,000), R-anti-ZIKV capsid (GeneTex; GTX133317, 1:1,000), M-anti-tubulin (Sigma-Aldrich; T5168, 1:1,000), anti-V5-tag mAb-HRP-DirecT (MBL; M215-7, 1:5,000), and M-anti-FlagM2-HRP (Sigma; A8592, 1:5000). For immunofluorescence microscopy, R-anti-calnexin (Cell Signaling Technology; 2433S, 1:200) and M-anti-J2 (Cell Signaling Technology; 76651L, 1:200) were used.

### Cell line generation

UFM1 KO Huh7 cells were generated by using a purified ribonucleoprotein complex consisting of Cas9 protein and single-guide RNAs (sgRNAs) targeting UFM1 synthesized by Synthego. Cas9 protein and sgRNAs were mixed at a ratio of 1:6 and then added to 1 × 10^6^ Huh7 cells in Neon Resuspension Buffer R, followed by electroporation using the Neon Transfection System (Invitrogen). Following recovery, single-cell clones were isolated and validated by anti-UFM1 immunoblot and genomic DNA sequencing, with one clone used here. Huh7-UFM1 KO cell pools overexpressing Flag-UFM1^WT^ or Flag-UFM1^∆C3^ were generated by lentiviral transduction, as previously discussed (62).

### Focus-forming assay for viral titer

Focus-forming assays were performed similarly to previously described for extracellular titer (63). Briefly, supernatants were harvested from ZIKV or DENV-infected cells 48 h after infection, serially diluted, and used to infect naïve Vero cells in triplicate wells of a 48-well plate for 3 h before overlay with methyl cellulose (Millipore Sigma, M0512). After 72 h, cells were washed with phosphate-buffered saline (PBS) and fixed with 1:1 methanol:acetone. Cells were blocked with 5% milk in phosphate-buffered saline with 0.1% Tween (PBS-T) and then immunostained with M-anti-4G2 antibody generated from the D1-4G2-4-15 hybridoma cell line against the flavivirus envelope protein (ATCC; 1:2,000). Infected cells were visualized following incubation with a horseradish peroxidase (HRP)-conjugated secondary antibody (1:500) and the VIP Peroxidase Substrate Kit (Vector Laboratories). The titer (focus-forming units per milliliter) was calculated from the average number of 4G2-positive foci at ×10 magnification, relative to the amount and dilution of virus used. For intracellular titer, cell pellets were washed in PBS and lysed through four freeze-thaw cycles using a dry ice/ethanol bath. Post-nuclear lysates were isolated by centrifugation and used to infect naïve Vero cells with a focus-forming assay performed as described above.

### Viral infections and generation of viral stocks

Infectious stocks of ZIKV-GLuc were generated by harvesting the supernatant 3–5 days post-transfection of pCDNA6.2 MR766 single intron NS1 GLuc flanking HDVr (33) into 293T cells. The viral stocks were titered on Vero cells as described above. ZIKV^Flag-NS2A^ virus (gift of Dr. Matthew J. Evans) was generated similarly to previously tagged NS2A viruses (43), with a 3XFlag cloned into the junction between NS1 and NS2A in the plasmid-based rescue system for ZIKV MR766 (44). Infectious stocks of a cell culture-adapted strain of genotype 2A JFH1 HCV (64) were generated and titered on Huh-7.5 cells by FFA, as described. DENV (dengue virus 2 Thailand/NGS-C/1944) (65), ZIKV (ZIKV/*Homo sapiens*/PRI/PRVABC59/2015), WNV (WNV strain 3000.0259 isolated in New York in 2000) (66), and yellow fever virus 17D (YFV-17D vaccine strain, gift of Dr. Helen Lazear) stocks were prepared in C6/36 cells and titered on Vero cells, as described above. For viral infections, cells were incubated in a low volume of DMEM containing the virus for 3–4 h, following which the infection media were replaced with cDMEM. The translation inhibitor cycloheximide (Sigma-Aldrich, 100 µM) and the flavivirus RNA-dependent RNA polymerase inhibitor MK-0608 (Aldrich, 50 µM) were added to cells during infection and were included in the replacement media when indicated.

### Quantification of the percentage of virally infected cells

Cells were immunostained for either orthoflavivirus envelope (M-anti-4G2, 1:1,000) or HCV NS5A (1:500, gift of Dr. Charles Rice), as well as nuclei (Hoescht). The percentage of infected cells was calculated as the number of viral antigen positive cells/the number of total cells (4G2 or NS5A/4′,6-diamidino-2-phenylindole [DAPI]) per field following imaging using a Cellomics ArrayScan VTI High Content Screening Reader (Duke Functional Genomics Facility). Values represent the mean ± standard error of the mean (*n* = 4 fields) from three independent experiments, with an average of 5,000 cells counted per experiment.

### RT-qPCR

Total cellular RNA was isolated using the RNeasy Mini kit (Qiagen) or TRIzol Reagent (Thermo Fisher). RNA was then reverse transcribed using the iSCRIPT cDNA synthesis kit (Bio-Rad) as per manufacturer’s instructions. The resulting cDNA was diluted 1:3 in nuclease-free distilled H2O. RT-qPCR was performed in triplicate using the Power SYBR Green PCR master mix (Thermo Fisher, Table 1) and the Applied Biosystems QuantStudio 6 Flex RT-PCR system. Viral copy number was measured in duplicate by RT-qPCR using the TaqMan Fast Virus 1-Step Mix (Thermo Fisher) with DENV or ZIKV specific probes (Table 1). The copy number of DENV or ZIKV was calculated by comparison to a standard curve of full-length *in vitro* transcribed DENV or ZIKV RNA.

**TABLE 1:** Oligonucleotides and probes used for RT-qPCR

Target	Forward primer (5′−3′)	Reverse primer (5′−3′)
*IFNB1*	CTTTGCTATTTTCAGACAAGATTCA	GCCAGGAGGTTCTCAACAAT
*IFNL1*	CTTCCAAGCCCACCACAACT	GGCCTCCAGGACCTTCAGC
*IFIT1*	TCCTTGGGTTCGTCTACAAAT	TTCTCAAAGTCAGCAGCCAGT
*UFL1*	AGCAAACAGGCCTCAACTGT	TTTCTGGTGCATCAGCTCAC
*RPL30*	ATGCTGTGCCCAGTTCAATA	ATTCTCGCTAACAACTGCCC
*DENV-NGC*	TCCCAAACGCAGTGATATTACAA	TGAGACCTTTGATCGTCAATGC
*DENV-NGC* probe	TGGTGTCCGTTTCCCCACTGCTCTT	
*ZIKV-PR*	CCGCTGCCCAACACAAG	CCACTAACGTTCTTTTGCAGACAT
*ZIKV-PR* probe	AGCCTACCTTGACAAGCAATCAGACACTCAA	

### *In vitro* transcription and electroporation of RNA

Plasmid DNA encoding a DENV replicon luciferase reporter (DENV-RLuc-SGR [8]) was linearized using XbaI (New England Biolabs). Purified linearized DNA was used as a template for *in vitro* transcription with the MEGAscript T7 transcription kit (Invitrogen). RNA was purified to be free of DNA and transfected in Huh7 cells via electroporation, as follows: 5 µg of RNA was mixed with 4 × 10^6^ Huh7 cells in Cytomix buffer (2 mM ATP, 10 mM K_2_HPO_4_, 0.15 mM CaCl_2_, 25 mM HEPES, 2 mM EGTA, 5 mM MgCL_2_, 120 mM KCl, and 5 mM glutathione) and electroporated at 27 V and 975 µF with a Gene Pulser XCell System (Bio-Rad). At 4 h post-electroporation, cells were washed with PBS, and cDMEM was replaced.

### Transfection

DNA transfections were performed using FuGENE6 (Promega) or PEIpro transfection reagent (Polyplus). The following siRNAs were used in this study: UFL1 (Qiagen-SI04371318), UFBP1 (Thermo Fisher-s35323), UBA5 (Qiagen-SI04146989), UFC1 (Qiagen-SI00755230), UFM1 (Horizon-L-021005-00-0005) or non-targeting AllStars negative control siRNA (Qiagen-1027280). siRNA transfection (30 pmol of siRNA, final concentration of 0.015 µM) was done using Lipofectamine RNAiMax (Invitrogen), with media changed 4 h after transfection.

### Luciferase assay

Luciferase activity of GLuc or RLuc was measured using the *Renilla* Luciferase Assay System (Promega, E2810). Briefly, cell supernatant or cell lysate was collected in 1× *Renilla* luciferase lysis buffer. *Renilla* luciferase assay reagent was prepared by adding 1 vol of 100× *Renilla* luciferase substrate to 100 vol of *Renilla* luciferase assay buffer. Cell lysate or supernatant (20–50 µL) was plated into an opaque 96-well plate, and 100 µL of *Renilla* luciferase assay substrate was dispensed into each well. Luciferase was read using a BioTek Synergy2 microplate reader.

### Immunoblotting

Cells were lysed in a modified radioimmunoprecipitation assay buffer (TX-100-RIPA) (50 mM Tris [pH 7.5], 150 mM NaCl, 5 mM EDTA, 0.1% SDS, 0.5% sodium deoxycholate, and 1% Triton X-100) or NP40 lysis buffer (20 mM Tris [pH 7.4], 100 mM NaCl, and 0.5% Nonidet P-40) supplemented with protease inhibitor (Sigma-Aldrich) and Halt phosphatase inhibitor (Thermo Fisher) at 1:100, and post-nuclear lysates were isolated by centrifugation. Quantified protein, as determined by Bradford assay (Bio-Rad), was resolved by SDS/polyacrylamide gel electrophoresis (PAGE) and transferred to nitrocellulose or polyvinylidene difluoride membrane membranes in the Trans-Blot Turbo buffer (Bio-Rad) using the Turbo Transfer system (Bio-Rad). Membranes were stained with Revert Total Protein Stain (Licor Biosciences) and blocked with 3% bovine serum albumin in PBS-T. Membranes were probed with primary antibodies directed against proteins of interest, washed with PBS-T, incubated with species-specific HRP-conjugated antibodies (Jackson ImmunoResearch; 1:5,000), or fluorescent secondaries (Licor Biosciences, 1:5,000), washed again with PBS-T, and treated with Clarity Western ECL substrate (Bio-Rad). Imaging was then performed using a LICOR Odyssey FC.

### Protein immunoprecipitation

Cells were lysed as above. Quantified protein (between 100 and 500 µg) was incubated with anti-V5 magnetic beads (Cell Signaling Technology) or anti-Flag magnetic beads (Sigma-Aldrich) in lysis buffer at room temperature for 45 minutes to 1 h with head-over-tail rotation. The beads were then washed 3× in PBS or PBS-T and eluted in 2× Laemmli buffer (Bio-Rad) with 5% 2-mercaptoethanol by incubating at 95°C for 5 minutes. Proteins were resolved by SDS/PAGE and immunoblotting as above.

### Immunofluorescence microscopy

Cells were fixed and permeabilized in 100% methanol and blocked with 10% FBS in PBS. Slides were stained with the indicated primary antibodies, washed 3× in PBS, incubated with conjugated Alexa Fluor secondary antibodies (Life Technologies), and mounted with ProLong Diamond + 4′,6-diamidino-2-phenylindole (Invitrogen). Imaging was performed on a Leica DM4B widefield fluorescence microscope using a 63× oil objective. All images were processed with National Institutes of Health Fiji/ImageJ.
